# WDR5 supports colon cancer cells by promoting methylation of H3K4 and suppressing DNA damage

**DOI:** 10.1186/s12885-018-4580-6

**Published:** 2018-06-20

**Authors:** Beth K. Neilsen, Binita Chakraborty, Jamie L. McCall, Danielle E. Frodyma, Richard L. Sleightholm, Kurt W. Fisher, Robert E. Lewis

**Affiliations:** 1 Eppley Institute, Fred & Pamela Buffett Cancer Center, University of Nebraska Medical Center, Omaha, NE 68198 USA; 2 Department of Pharmaceutical Sciences, University of Nebraska Medical Center, Omaha, NE 68198 USA; 3 Department of Pathology and Microbiology, University of Nebraska Medical Center, Omaha, NE 68198 USA; 4 Present address: Department of Pharmacology, Duke University School of Medicine, Durham, NC 27710 USA; 5 Present address: Department of Microbiology, Immunology, and Cell Biology, West Virginia University, Morgantown, WV 26506 USA

**Keywords:** WDR5, OICR-9429, H3K4Me3, γH2AX, Colon cancer

## Abstract

**Background:**

KMT2/MLL proteins are commonly overexpressed or mutated in cancer and have been shown to support cancer maintenance. These proteins are responsible for methylating histone 3 at lysine 4 and promoting transcription and DNA synthesis; however, they are inactive outside of a multi-protein complex that requires WDR5. WDR5 has been implicated in cancer for its role in the COMPASS complex and its interaction with Myc; however, the role of WDR5 in colon cancer has not yet been elucidated.

**Methods:**

WDR5 expression was evaluated using RT-qPCR and western blot analysis. Cell viability and colony forming assays were utilized to evaluate the effects of WDR5 depletion or inhibition in colon cancer cells. Downstream effects of WDR5 depletion and inhibition were observed by western blot.

**Results:**

WDR5 is overexpressed in colon tumors and colon cancer cell lines at the mRNA and protein level. WDR5 depletion reduces cell viability in HCT116, LoVo, RKO, HCT15, SW480, SW620, and T84 colon cancer cells. Inhibition of the WDR5:KMT2/MLL interaction using OICR-9429 reduces cell viability in the same panel of cell lines albeit not to the same extent as RNAi-mediated WDR5 depletion. WDR5 depletion reduced H3K4Me3 and increased phosphorylation of H2AX in HCT116, SW620, and RKO colon cancer cells; however, OICR-9429 treatment did not recapitulate these effects in all cell lines potentially explaining the reduced toxicity of OICR-9429 treatment as compared to WDR5 depletion. WDR5 depletion also sensitized colon cancer cells to radiation-induced DNA damage.

**Conclusions:**

These data demonstrate a clear role for WDR5 in colon cancer and future studies should examine its potential to serve as a therapeutic target in cancer. Additional studies are needed to fully elucidate if the requirement for WDR5 is independent of or consistent with its role within the COMPASS complex. OICR-9429 treatment was particularly toxic to SW620 and T84 colon cancer cells, two cell lines without mutations in WDR5 and KMT2/MLL proteins suggesting COMPASS complex inhibition may be particularly effective in tumors lacking KMT2 mutations. Additionally, the ability of WDR5 depletion to amplify the toxic effects of radiation presents the possibility of targeting WDR5 to sensitize cells to DNA-damaging therapies.

**Electronic supplementary material:**

The online version of this article (10.1186/s12885-018-4580-6) contains supplementary material, which is available to authorized users.

## Background

Recent technological advances and significant efforts to identify genetic alterations in cancer demonstrate that the KMT2/MLL proteins are commonly altered in multiple cancers. While it is well-established that KMT2A/MLL1 is commonly involved in pro-tumorigenic chromosomal translocations or rearrangement in leukemia [1], the frequency of KMT2/MLL genetic alterations and overexpression in other tumor types, including breast, prostate, pancreas, stomach, and colon, was surprising.

The KMT2/MLL family of proteins includes KMT2A/MLL1, KMT2B/MLL2 or MLL4, KMT2C/MLL3, KMT2D/MLL4 or MLL2, KMT2F/SETD1A, and KMT2G/SETD1B. KMT2/MLL family proteins, while highly related, have both distinct and redundant functions [2]. In general, the KMT2/MLL proteins are the major components of the SET/MLL COMplex of Proteins Associated with Set1 (COMPASS) complex in humans that is responsible for mono-, di-, and tri- methylating histone H3 at lysine 4 (H3K4). In humans, the KMT2/MLL proteins have little methylation activity outside of the SET/MLL COMPASS complex, which consists of one of the KMT2/MLL proteins (MLL1, MLL2, MLL3, MLL4, SETD1A, or SETD1B) in addition to a common subcomplex that includes WDR5, RBBP5, ASH2L, DPY30 (WRAD subcomplex) [3].

The formation of this complex stimulates the KMT2/MLL activity by increasing H3K4 affinity [4]. The addition of methyl groups to H3K4 generally promotes transcription by recruiting transcription factors and coactivators to promoters while also interfering with the addition of epigenetic modifications that repress transcription [4]. However, the location of methylation (in promoters or enhancers) and degree of methylation (mono-, di-, and tri-methylation) varies between KMT2/MLL proteins and can be tissue-specific.

Recent studies have identified a correlation between H3K4Me3 enrichment and transcriptional fidelity as well as enhanced elongation rates [5, 6] suggesting a potential role for the COMPASS complex in promoting DNA synthesis and preventing DNA damage during replication thereby supporting cancer cell proliferation. MLL1 and WDR5 have been shown to be required for proper chromosome congression and spindle assembly during mitosis, which may affect chromosomal stability [7]. Additionally, mutations in MLL2 have been shown to cause genome instability [8]. In another report, AML driven by KMT2A/MLL1 fusions were shown to be proficient in DNA damage response (DDR) leading to resistance to PARP inhibitors. However, depleting, or inhibiting cells of the KMT2A/MLL1 downstream target HOXA9 caused DDR impairment and PARP inhibitor sensitization [9]. Together these data suggest a role for this complex in supporting DNA replication and maintaining DNA fidelity, thereby promoting cancer cell survival and proliferation. Consistent with this proposed role by which WDR5 may support tumor growth and survival, depletion of KMT2D in multiple pancreatic cancer cell lines increased their responsiveness to 5-FU [10] suggesting the possibility that KMT2/MLL inhibitors could be used for chemotherapy or radiation sensitization.

Within cancer, the COMPASS complex has been shown to promote transcriptional reprogramming through increased methylation at H3K4 [11] and by interacting with commonly recognized oncogenic transcription factors. Specific targets of KMT2/MLL epigenetic regulation have been shown to include hTERT (KMT2A, in melanoma) [12], several HOX genes (KMT2A) [9], ERalpha target genes in breast cancer (KMT2D) [13, 14], and androgen receptor target genes in prostate cancer (MLL1 and WDR5) [15]. Specifically, KMT2C and KMT2D depletion caused downregulation of genes related to cell-cycle and proliferation based on microarray and gene-set enrichment analysis [10]. In these reports, inhibition or depletion of key KMT2/MLL components decreased the expression of important transcriptional targets thereby inhibiting cancer cell growth [13, 16]. In colon cancer, KMT2D and KMT2C mutations are common and are present in 10% of tumors (Table 1, COSMIC v83). In contrast, the common components of the COMPASS complex were rarely mutated (Table 1). Additionally, many of the commonly used colon cancer cell lines harbor multiple mutations within KMT2/MLL family members (Additional file 1: Table S1, COSMIC Cell Lines Project, [17]). The effects of these mutations are still being debated, but are likely pro-tumorigenic. One study demonstrated that KMT2D promoted global H3K4 monomethylation in transcriptional enhancers, and depletion of KMT2D in two colon cancer cell lines (HCT116 and DLD-1) decreased cancer cell proliferation and migration [11].

**Table 1:** Percent of samples with mutated COMPASS complex proteins (COSMIC v83)

Frequency of Mutations in Colon Adenocarcinoma		
Gene	Percent Mutated	Samples (Mutated/Tested)
KMT2C/MLL3	13%	323/2478
KMT2D/MLL2	11%	243/2209
KMT2A/MLL	7%	152/2178
KMT2B/MLL4	7%	150/2130
KMT2F/SETD1A	6%	116/2109
KMT2G/SETD1B	3%	67/2098
RBBP5	2%	32/2109
WDR5	1%	28/2109
ASH2L	1%	25/2109
DPY30	< 1%	6/2098

Based on the significant requirement for the WRAD subcomplex for activity of all KMT2/MLL proteins and the emerging evidence that KMT2/MLL proteins likely play a role in tumor maintenance, evaluating the efficacy of targeting components of the WRAD subcomplex for the treatment of cancer could be highly efficacious.

In this report, we show that WDR5, a common component of the SET/MLL COMPASS complex, is overexpressed in human colon cancer tumors and cell lines and is required for colon tumor cell proliferation. WDR5 depletion decreased H3K4Me3 and increased DNA damage as measured by increased H2AX phosphorylation. WDR5 depletion sensitized cells to ionizing radiation further as the combination increased DNA damage and PARP cleavage. Further, we show that OICR-9429, an inhibitor of the interaction between KMT2/MLL and WDR5, is required for colon cancer growth. Thus, these data demonstrate a previously unrecognized role for WDR5 in colon cancer cell proliferation and survival.

## Methods

### Cell culture

Colon cancer cell lines HCT116 (ATCC CCL-247), LoVo (ATCC CCL-229), RKO (CRL-2577), HCT15 (ATCC CCL-225), SW480 (ATCC CCL-228), SW620 (ATCC CCL-227), and T84 (ATCC CCL-248) were obtained from American Type Culture Collection (ATCC). Cells were grown in Dulbecco’s Modified Eagle’s Medium (DMEM) with 10% fetal bovine serum (FBS). All colon cancer cells were grown with ambient O_2_ and 5% CO_2_ at 37 °C. Immortalized, non-transformed human colonic epithelial cell lines (HCEC) were kindly provided by Jerry Shay (UT Southwestern) [18]. HCEC media consists of four parts DMEM to one-part media 199 (Sigma-Aldrich) supplemented with 1 μg/mL hydrocortisone, 25 ng/mL EGF, 10 μg/mL insulin, 5 nM sodium selenite, 2 μg/mL transferrin and 2% cosmic calf serum (GE Healthcare). HCECs were grown in 2% O_2_ and 5% CO_2_ at 37 °C within an enclosed hypoxia chamber. HCECs are grown on Corning™, Primaria™ plates.

### siRNA transfections

Pooled or individual ON-TARGET plus siRNAs targeting WDR5 (L-013383-00-0005) or a non-targeting siRNA control (D-001810-01) (Dharmacon), were transfected into the cell lines listed above using the Lipofectamine RNAiMAX (Invitrogen) reverse transfection protocol and as described following: 5 μL of RNAiMax, 2 mL of media (150,000 cells/mL), 500 μL Opti-MEM media, and 40 nM RNAi were combined in 6-well plates. The same reverse transfection protocol was utilized for HCEC transfections with the following reagent quantities: 5 μL RNAiMax transfection reagent per 5 mL of media and 100,000 cells/mL with an RNAi concentration of 20 nM in 6-well plates. RIPA lysis buffer with protease and phosphatase inhibitors was used to lyse cells 72 h after transfected unless otherwise noted. siRNA sequences can be found in Additional file 1: Table S2.

### Propidium iodide (PI) stain cell cycle analysis

The sub-G1 peak was measured following propidium iodide (PI) staining to assess cell apoptosis. All adherent and nonadherent cells were collected and placed in round bottom 12 × 75 mm polystyrene tubes (BD Falcon, 352,054). Centrifugation for 3 min at 2800 RPM using an Immunofuge II was completed to pellet the cells. The media was aspirated, and the cells were resuspended in PBS. Cells were again pelleted by centrifugation for 3 min at 2800 RPM using an Immunofuge II. The PBS was aspirated, and the cells were fixed in 1 mL of ice cold 70% ethanol overnight at − 20 °C. Cells were warmed to room temperature, pelleted by centrifugation, then rehydrated in room temperature PBS and incubated for 15 min at 37 °C. Centrifugation was utilized to pellet the cells, PBS was aspirated, and the cells were resuspended in PI stain for 8–15 h (overnight). PI staining was evaluated using an LSR II flow cytometer and analyzed using FlowJo Cell Cycle analysis.

### Annexin V/Propidium iodide (PI) apoptosis analysis

Cells were assayed for apoptosis based on Annexin V/PI staining using an Invitrogen FITC Annexin V/Dead Cell Apoptosis Kit (V13242) following the manufacturer’s instructions. All nonadherent and adherent cells were collected in a 15 mL conical. Centrifugation was used to pellet the cells. The media was aspirated, cells were resuspended in PBS, and counted using a hemocytometer. 200,000 cells were placed in a 12 × 75 mm round bottom polystyrene tube (BD Falcon, 352,054) and again centrifuged to pellet the cells. The PBS was aspirated, and the cells were resuspended in 100 μL of 1X Binding buffer at a concentration of 2 million cells/mL (200,000 cells in 100 μL of 1X Binding buffer). 5 μL of Annexin V solution and 1 μL of PI were added to each sample and allowed to incubate for 15 min. Then 400 μL of 1X Binding buffer was added and samples were put on ice. Staining was evaluated using a Becton-Dickinson LSR II flow cytometer immediately after staining. Results were analyzed using FlowJo software to determine the percentage of cells that stained with Annexin V (early apoptosis), PI (late apoptosis), or both (necrosis).

### Radiation treatment

200,000 cells/well were transfected on 6-well plates. Transfections were done as described above. At 48 h, 3 Gray of ionizing radiation was applied to the cells in a single dose (RS-2000 Irradiator). At 96 h after plating, cells were collected for western blot analysis.

### Reagents

OICR-9429 was purchased from Caymen Chemical (1801787–56-3). DMSO was purchased from Fisher (D128–500). OICR-9429 was dissolved in DMSO at a stock concentration of 10 mM. Stock OICR-9429 (10 mM) or DMSO was dissolved in pre-warmed media at a 1:1000 ratio to achieve a final drug concentration of 0.1% DMSO or 10 μM of OICR-9429 for drug treatments [19].

### Cell growth assay

5000–10,000 (HCEC, LoVo, T84) cells/well were transfected or plated on white or clear 96-well plates. Reverse transfections followed the same protocol as previously described but were completed using a 1:25 ratio for all the reagents (20 μL of the final mixture added to each well). At 48, 72, or 96 (start with half as many cells) hours post-transfection or drug treatment, alamarBlue® (ThermoFisher Scientific) was added at a ratio of 100 μL per 1 mL of media to each well. Plates were incubated at 37 °C for 1–3 h and fluorescence was measured (POLARstar OPTIMA). Results were background subtracted (well with media + alamarBlue® without any cells) and normalized with the control being set to 1. In other instances, cell viability was measured using the manufacturers’ protocol with the CellTiter-Glo® Luminescent Cell Viability Assay (Promega). Based on this protocol, 90 μl of CellTiter-Glo® reagent was added to each well, cells were then shaken for two minutes, and incubated for 10 min at room temperature to stabilize the signal. The luminescence was measured using a POLARstar OPTIMA. Note: CellTiter-Glo® must be completed on plates with opaque side walls.

### Western blot analyses

Radioimmunoprecipitation assay (RIPA) buffer (50 mM Tris-HCl, 1% NP-40, 0.5% Na deoxycholate, 0.1% Na dodecyl sulfate, 150 mM NaCl, 0.5 mM Na_3_VO_4_, 2 mM EDTA, 2 mM EGTA, 10 mM NaF, 10 μg/mL aprotinin, 20 mM leupeptin, 2 mM PMSF) was used to prepare whole cell lysate from collected cells. Promega BCA protein assay was utilized to evaluate protein concentration. SDS-PAGE gel electrophoresis was completed, proteins were transferred to nitrocellulose membranes, membranes were blocked for 45 min in PBS-based blocking buffer (LI-COR Biosciences, 927–40,000), and incubated in primary antibody (listed below) at 4 °C overnight. Secondary antibodies (LICOR IRDye 680LT and 800CW) were diluted 1:10,000 in 0.1% TBS-Tween. The LI-COR Odyssey was used to image the western blots.

### Antibodies

Primary antibodies (catalog numbers) and dilutions were as follows:

WDR5 (ab22512, Abcam) 1:1000; α-tubulin (B-5-1-2, Santa Cruz) 1:2500; PARP (9542, Cell Signaling) 1:1000; Phospho-Histone H2A.X (Ser139)(2577, Cell Signaling); H2A.X (2595, Cell Signaling); H3K4Me3 (ab8580, Abcam) 1:1000; H3K4Me1 (ab8895, Abcam) 1:1000; Histone 3 (ab1791, Abcam) 1:2500; and p53 (6243, Santa Cruz) 1:1000.

### RT-qPCR

1 mL TriReagent (MRC, TR118) was used to collect RNA, which was then stored at − 80°C until extraction. The manufacturer’s protocol was followed for RNA extraction. A NanoDrop 2000 (Thermo Scientific) was used to quantify the RNA. Following the manufacturer’s protocols, reverse transcription was completed using iScript™ Reverse Transcription Supermix (Bio-Rad, 170–8840) with 1 μg of total RNA in 20 μL reaction volume. Amplification and quantification was performed using the SsoAdvanced™ Universal SYBR Green Supermix (Bio-Rad). Primer sequences and reaction conditions for RT-qPCR are listed in Additional file 1: Table S3.

*TCGA.*

The FPKM-UQ normalized RNASeq values of primary tumors (*n* = 478 with 456 unique patients) and normal solid tissue (*n* = 41) samples from within The Cancer Genome Atlas (TCGA) Colon Adenocarcinoma (COAD) dataset was used to evaluated mRNA expression. Results were analyzed for statistical significance using an unpaired Student’s *t* tests.

### Statistical analyses

**Fig. 1:**
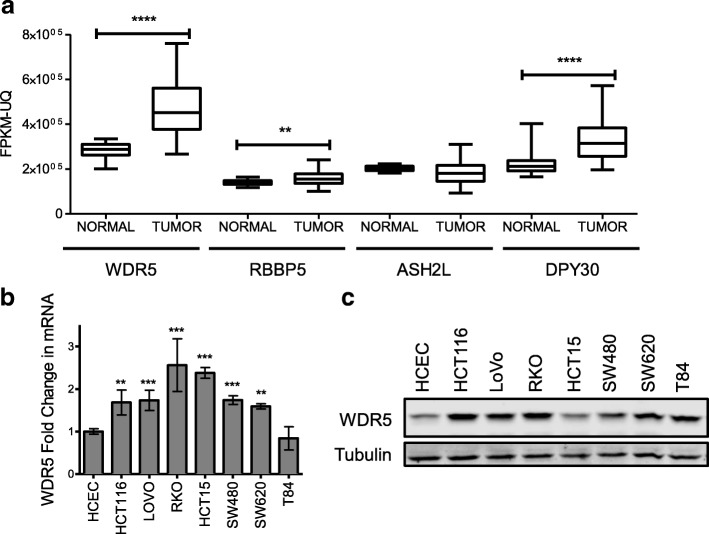
WDR5 is overexpressed in colon cancer cells. **a** WDR5, RBBP5, ASH2L, and DPY30 gene expression (RNASeq) data from the Colon Adenocarcinoma (COAD) dataset within TCGA for unpaired primary colon tumors and normal solid tissue samples. Tumor includes 478 samples from 456 patients for each gene. Normal includes 41 samples from 41 patients for each gene. For each boxplot the middle line represents the median, the box represents the 25th to 75th percentile and the whiskers represent the 5th to 95th percentile. The results published here are in whole or part based upon data generated by the TCGA Research Network: http://cancergenome.nih.gov/. **b** RT-qPCR and (**c**) western blot of WDR5 in a panel of colon tumor cell lines as compared to immortalized, non-transformed HCECs. RT-qPCR data are shown as mean ± SD. ** *p* < 0.01 *** *p* < 0.001 **** *p* < 0.0001

**Fig. 2:**
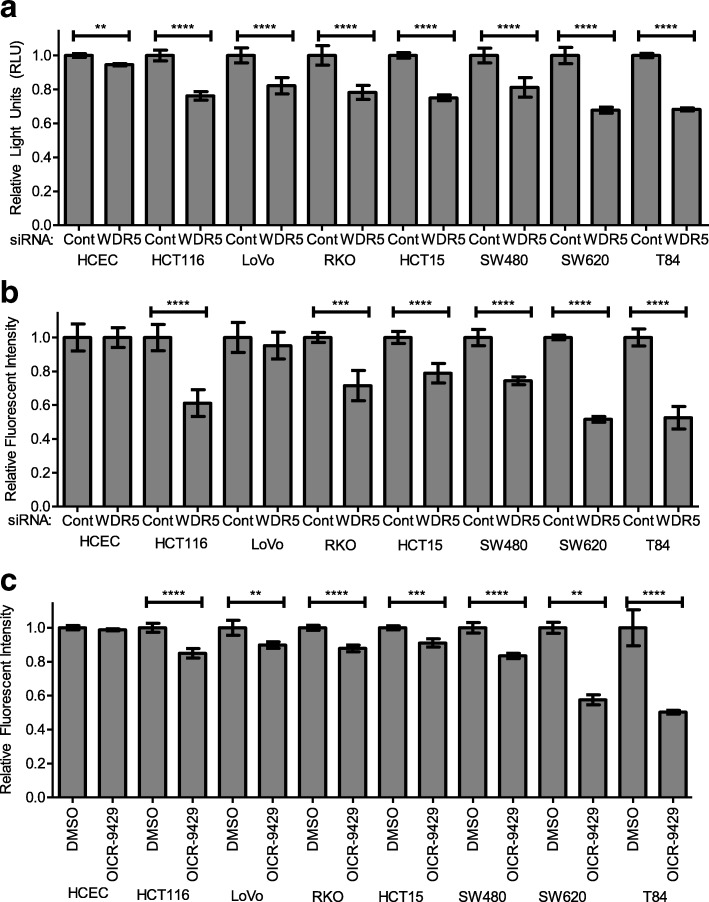
WDR5 depletion or disruption of the COMPASS complex limits cell proliferation or viability in colon cancer cells. **a** and **b** Cell viability in a panel of colon cancer cells as compared to HCECs following RNAi-mediated depletion of WDR5. Viability was measured by CellTiter-Glo® (**a**) and alamarBlue® (**b**) assays 72 h after transfection. **c** Cell viability in a panel of colon cancer cells as compared to HCECs following 72-h treatment with 10 μM OICR-9429 as measured by alamarBlue®. Data are shown as relative light units or relative fluorescent intensity ± SD. ** p < 0.01 *** p < 0.001 **** p < 0.0001

**Fig. 3:**
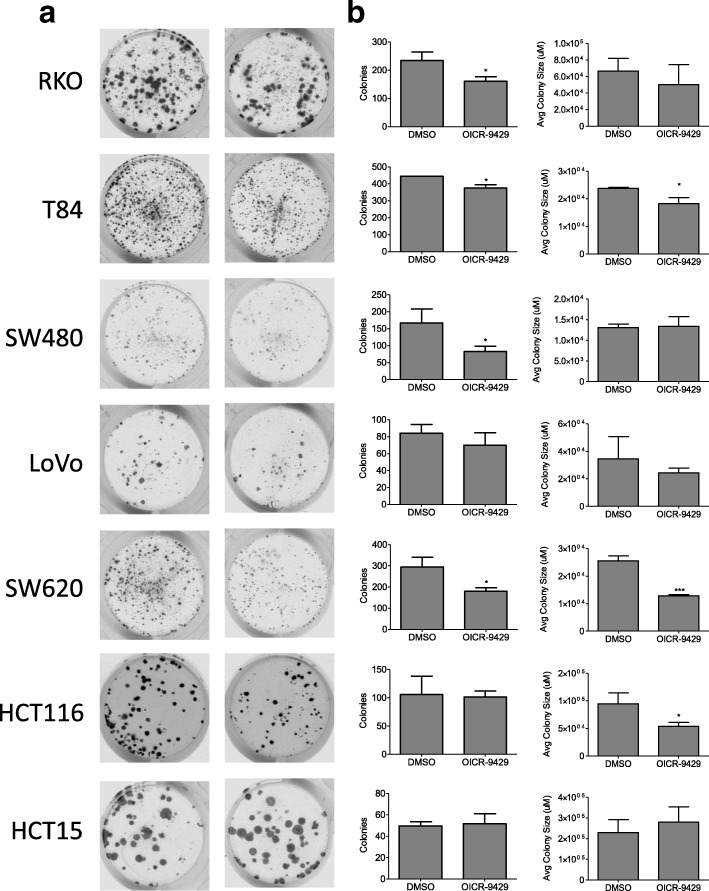
Disruption of the COMPASS complex decreases cell colonies in colon cancer cells. **a** and **b** Representative pictures (**a**) and quantification of number and average size of colonies (**b**) formed on 24-well plates in colon cancer cell lines following treatment with OICR-9429 treatment for 10–14 days. Number of colonies and average colony size are shown as mean ± SD. * *p* < 0.05 ** p < 0.01 *** p < 0.001

**Fig. 4:**
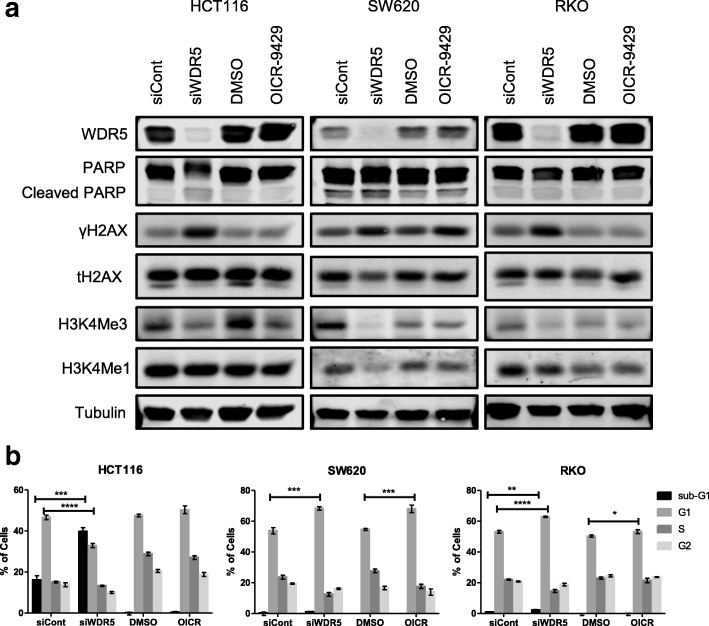
WDR5 depletion increases DNA damage and reduces H3K4Me3. **a** Western blot of PARP, γH2AX, total H2AX, H3K4Me3, and H3K4Me1 following 96-h WDR5 knockdown or 72-h OICR-9429 treatment in colon cancer cells. **b** Percentage of cells within the sub-G1, G1, S, or G2 phase based on propidium iodide staining and flow cytometry analysis following WDR5 depletion for 72 h or 10 μM OICR-9429 treatment for 48 h in three colon cancer cell lines

Prism Software (GraphPad, La Jolla, CA) was used to calculate P and EC50 values. *P* values of less than or equal to 0.05 were considered statistically significant. Significance of qPCR results was evaluated using one-way ANOVA with Dunnett’s post-test to individually compare all cell lines to the control cell line HCEC (Fig. 1b). The TCGA COAD RNASeq FPKM-UQ expression, cell viability assays, colony number and size, Annexin V/PI apoptotic assay (early and late apoptosis), and Propidium Iodide cell cycle analysis (sub-G1 peak and G1 phase) were statistically evaluated using an unpaired, two-sided t-test for each target (Fig. 1a), cell line analyzed (Figs. 2 and 3b), and treatment (Fig. 4b). Data are shown as mean +/− standard deviation (SD) unless otherwise noted.

## Results

### WDR5 is overexpressed in colon cancer cells

To evaluate the expression of the components of the WRAD subcomplex in cancer, the mRNA levels of WDR5, RBBP5, ASH2L, and DPY30 in tumors compared to normal solid tissue samples were examined based on RNASeq analysis from the colon adenocarcinoma (COAD) dataset within The Cancer Genome Atlas (Fig. 1a). WDR5, RBBP5, and DPY30 are increased in tumors relative to normal tissue; however, WDR5 is expressed at the highest level and shows the most dramatic increase in expression between normal tissue and colon tumor tissue so it was selected for further study. WDR5 is also overexpressed at the mRNA (Fig. 1b) and protein level (Fig. 1c) in a panel of colon cancer cells as compared to immortalized, yet non-transformed human colon epithelial cells (HCECs) [18] suggesting WDR5 may play a pro-tumorigenic role in colon cancer.

### Validation of four siRNAs targeting WDR5

To evaluate the importance of WDR5 in colorectal cancer, cell viability following RNAi-mediated WDR5 depletion was measured. However, prior to performing this analysis, the individual siRNA oligos targeting WDR5 were validated. Evaluation of the individual oligos from the SMARTpool (Dharmacon) of four oligos targeting WDR5 revealed that all four dramatically decreased WDR5 levels. However, in HCT116 cells, oligo #6, the SMARTpool (a pre-mixed pool of all four oligos), and 1:1:1:1 pool of all four oligos dramatically decreased viability to a level substantially lower than the other three individual oligos (#5, #7, and #8) even though the levels of WDR5 depletion were comparable (Additional file 1: Figure S1A). Visually, oligo #6 and pools containing all four oligos induced substantial cell death and cell non-adherence, whereas oligos #5, #7, and #8 appeared to reduce proliferation and induced a lower level of cell death. Examining the mechanism further, multiple oligos induced DNA damage, as evidenced by increased phosphorylation of H2AX (γH2AX), but only oligo #6 increased p53 expression and induced PARP cleavage in HCT116 cells (Additional file 1: Figure S1B). This observation suggested an additional off-target effect for oligo #6 distinct from its ability to suppress the expression of WDR5. A blast search using the oligo #6 sequence demonstrated a 100% match to WDR5, but also shared a high degree of similarity to ME1 sharing a 14-nucleotide substring within the 19-nucleotide siRNA oligo (Additional file 1: Figure S1C). Previously, ME1 depletion was shown to induce p53 expression [20], suggesting this off-target effect could cause p53 induction in HCT116 cells. Reassuringly, all four individual oligos and both pools reduced HCT116 viability as measured by alamarBlue® following WDR5 depletion by more than 30% in 72 h suggesting WDR5 itself is playing a role supporting colon cancer cells. However, to avoid the possibility of confounding off-target effects and non-specific p53 induction, oligo #6 was excluded from the oligo pools in subsequent experiments.

### WDR5 is required for cancer cell survival

The importance of WDR5 in colorectal cancer is suggested by its selective upregulation in colon tumor cells and tissues compared to normal colonic epithelium. To determine whether WDR5 is required for colon cancer cell survival, cell viability in colon cancer cell lines and HCECs following transient WDR5 depletion by RNAi was measured. Cell viability was measured using CellTiter-Glo® Luminescent Cell Viability Assay 72 h after WDR5 depletion. WDR5 depletion reduced cell ATP levels by 15–30% in six colon cancer cell lines (Fig. 2a). These results were largely confirmed using the alamarBlue® Cell Viability Assay after 96 h of WDR5 depletion (Fig. 2b) with the only change being WDR5 depletion having no effect on viability in LoVo cells as measured by alamarBlue®. In contrast to the colon cancer cell lines, following WDR5 depletion HCECs demonstrated only a 5% decrease in cell ATP levels (Fig. 2a) and no difference in viability as measured by the alamarBlue® assay (Fig. 2b).

To evaluate the effect of WDR5 inhibition, the effect of OICR-9429 treatment on colon cancer cells and HCECs was examined. OICR-9429 is an antagonist of the interaction of WDR5 with peptide regions of KMT2/MLL and Histone 3, and disrupts COMPASS complex formation by blocking the interaction between WDR5, KMT2/MLL, and RBBP5 [19, 21]. Previous reports have also demonstrated that treatment with 10 μM OICR-9429 disrupted the interaction of WDR5 with MLL1 or RBBP5 to less than 20% based on co-immunoprecipitation experiments, and treatment with 5–20 μM OICR-9429 dramatically decreased cell viability in in vitro models of AML [19]. The effect of OICR-9429 on HCT116 colon cancer cells was evaluated by performing a dose response curve based on cell viability as measured with alamarBlue® following treatment with OICR-9429 for 48 h with doses ranging from 10 nM to 100 μM (Additional file 1: Figure S2) which revealed that 10 μM OICR-9429 substantially decreased cell viability. Therefore, based on previous reports demonstrating substantial interference in COMPASS complex formation and the drug dose response curve results, a dose of 10 μM was selected for future studies.

Treatment with 10 μM OICR-9429 for 72 h also decreased cell viability (alamarBlue® Cell Viability Assay), but to a lesser extent than seen with WDR5 depletion in some colon cancer cell lines (Fig. 2c). Interestingly, OICR-9429 treatment had less of an effect in RKO and HCT116 cells, two cell lines that harbor WDR5 mutations that may reduce the affinity of OICR-9429 for WDR5. Two cell lines with wildtype WDR5 and relatively few or no mutations in other COMPASS components (Additional file 1: Table S1), SW620 and T84 cells, were more sensitive to both WDR5 depletion as well as OICR-9429 treatment with approximately a 50% decrease in cell viability over 72 h.

### OICR-9429 treatment dramatically decreases colony growth in colon cancer cell lines

Based on the known contribution of WDR5 to the COMPASS complex that methylates lysine 4 on histone 3 (H3K4), we hypothesized that the effects of WDR5 depletion or OICR-9429 treatment will be enhanced over time. Therefore, the effect of OICR-9429 on colony growth of colon cancer cell lines was examined (Fig. 3a). OICR-9429 treatment decreased the number of colonies in RKO, T84, SW480, and SW620 cells, with a downward trend seen in LoVo cells (Fig. 3b). Additionally, colony size was decreased in T84, SW620, and HCT116 cells, while RKO and LoVo cells trended downwards (Fig. 3b).

### WDR5 depletion increases DNA damage and decreases trimethylation of H3K4

To further examine the role WDR5 plays in cancer, the effect of WDR5 depletion (oligos #7 and #8 only) and OICR-9429 treatment on H3K4Me3, H3K4Me1, and phosphorylation of H2AX (γH2AX) was examined in HCT116, SW620, and RKO cells. These cell lines were chosen because HCT116 cells were highly sensitive to WDR5 depletion, but much less so to OICR-9429 treatment; RKO cells were sensitive to both WDR5 depletion and OICR-9429 treatment, but to a lesser extent overall; and SW620 cells were highly sensitive to both WDR5 depletion and OICR-9429 treatment based on the cell viability assays (Fig. 2). In all three cell lines, WDR5 depletion induced γH2AX formation and decreased H3K4Me3 (Fig. 4a). In SW620 cells, WDR5 depletion also decreased H3K4Me1 (Fig. 4a). OICR-9429 treatment induced γH2AX in SW620 cells, but did not affect γH2AX in the other two cell lines (Fig. 4a). OICR-9429 treatment decreased H3K4Me3 levels in HCT116 cells and to a lesser extent in RKO and SW620 cells (Fig. 4a).

To evaluate the ability of WDR5 depletion or OICR-9429 treatment to induce apoptosis or affect cell cycle, Annexin V/Propidium Iodide (PI) Apoptosis staining and Propidium Iodide (PI) Cell Cycle analyses were completed and analyzed by flow cytometry. In HCT116 cells, WDR5 depletion (oligos #5, #7, and #8) for 72 h caused a robust induction of apoptosis as evidenced by a significant increase in cells in early apoptosis following Annexin V/PI staining (Additional file 1: Figure S4) and sub-G1 peak following PI cell cycle analysis (Fig. 4b, Additional file 1: Figure S3). Treatment with 10 μM OICR-9429 for 48 h also increased the percentage of HCT116 cells in late apoptosis (Additional file 1: Figure S4); however, OICR-9429 treatment induced apoptosis to a lesser extent than WDR5 depletion (Fig. 4b, Additional file 1: Figure S4). This is consistent with the effect of these two treatments on cell viability that demonstrated a substantial decrease in viability following WDR5 depletion and smaller effect of OICR-9429 treatment in HCT116 cells (Fig. 2). In SW620 cells, there were only slight increases in early apoptotic cells (Additional file 1: Figure S4), but the percentage of cells within the G1 phase was substantially increased for both WDR5 depletion (oligos #5, #7, and #8) for 72 h or 10 μM OICR-9429 treatment for 48 h (Fig. 4b, Additional file 1: Figure S3). This is consistent with the viability data in SW620 cells (Fig. 2), which demonstrated a consistent robust decrease of cell viability or cell number following WDR5 depletion or OICR-9429 treatment. RKO cells demonstrated a minor detrimental effect on viability following either WDR5 depletion (oligos #5, #7, and #8) for 72 h or 10 μM OICR-9429 treatment for 48 h based on only a small increase in the fraction of early apoptotic cells (Additional file 1: Figure S4). In RKO cells, cell cycle analysis also revealed a small increase in the percentage of cells within the G1 phase particularly with WDR5 depletion (Fig. 4b, Additional file 1: Figure S3).

These results suggest that WDR5 depletion induces DNA damage in colon cancer, but the OICR-9429 treatment is unable to fully replicate this effect in HCT116 and RKO colon cancer cells. This could be due to the presence of WDR5 mutations in these cell lines that render them less sensitive to OICR-9429 treatment. In contrast, SW620 cells that harbor wildtype WDR5 appear to be equally sensitive to WDR5 RNAi-mediated depletion and OICR-9429 treatment and demonstrate increased γH2AX with either manipulation. The effect on H3K4 methylation appears to be more consistently affected by OICR-9429 treatment. This could be due to the drug’s ability to inhibit the COMPSS complex independent of WDR5. This raises a question as to whether the effect of WDR5 on γH2AX is a function of its role within the COMPASS complex or another mechanism. In fact, RBBP5 depletion did not affect cell viability suggesting that WDR5 may function outside of the COMPASS complex to promote tumorigenesis (Additional file 1: Figure S5).

### WDR5 depletion sensitizes colon cancer cells to radiation-induced DNA damage

**Fig. 5:**
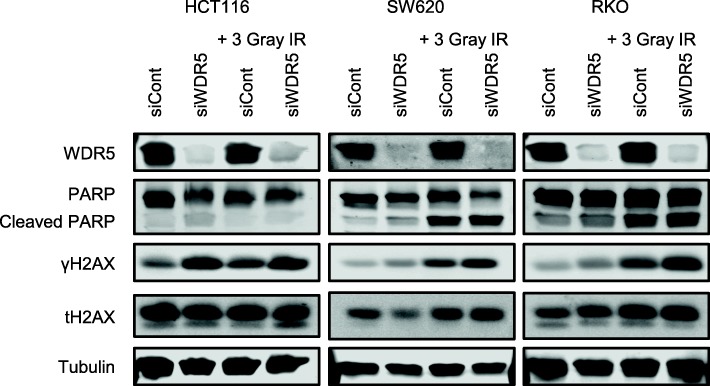
WDR5 depletion increases sensitivity to irradiation. Western blot of γH2AX and PARP following 96-h WDR5 knockdown with the addition of a single dose of 3 Gray ionizing radiation 48 h prior to collection

To evaluate the extent to which loss of WDR5 sensitizes cells to DNA damage, the effect of WDR5 depletion (oligos #7 and #8 only) on radiation-induced γH2AX formation and PARP cleavage was assessed. HCT116, SW620, and RKO cells were depleted of WDR5 for 48 h prior to irradiation. Cells were allowed to recover for 48 h before collection. In cells transfected with a non-targeting siRNA, irradiation increased γH2AX levels. This radiation-induced increase in γH2AX levels was further amplified with the loss of WDR5. SW620 and RKO cells demonstrated a step-wise increase in γH2AX levels with WDR5 depletion and irradiation with maximal γH2AX in the cells that received irradiation in conjunction with WDR5 depletion (Fig. 5). In contrast, HCT116 cells demonstrated a substantial increase in γH2AX with WDR5 depletion regardless of the addition of radiation (Fig. 5). This could be a consequence of the high level of endogenous genomic instability in HCT116 cells. Regardless, in all conditions, WDR5 depletion increased γH2AX levels that indicate increased DNA damage.

## Discussion

WDR5 functions to serve as a core component of several complexes within the cell [22]. It has been studied most for its role in the SET/MLL COMPASS complex, which mono-, di-, and tri-methylates histone 3 lysine 4 (H3K4Me1–3) [23–25]. WDR5 has also been shown to contribute to specific recognition of H3K4Me3 targets [26] contributing to increased transcription of target genes as H3K4 methylation often occurs within enhancer or promoter regions depending on the KMT2/MLL protein included in the complex. As part of the COMPASS complex, WDR5 has a significant role in development as it regulates embryonic stem cell pluripotency, self-renewal, and transcriptional reprogramming. The developmental effects of WDR5 are largely a consequence of its ability to modulate transcription of specific targets, including multiple HOX genes and SOX9 [27–30] that promote stem cell-like states by promoting the maintenance of active chromatin for pluripotency genes [28, 31–34].

WDR5 has also been shown to promote its own expression through a positive feedback loop where increased H3K4Me3 at the WDR5 promoter increases its transcription [30]. This positive feedback loop could be contributing to the consistent overexpression of WDR5 demonstrated here in both colon cancer cell lines as well as human colon tumors; however, this is difficult to definitively demonstrate experimentally. The overexpression of WDR5 is not unique to colon cancer as recent studies have demonstrated WDR5 is overexpressed in several cancer types including breast, prostate, bladder, and pancreatic cancer. WDR5 overexpression has been clinically associated with worse patient outcomes in breast cancer and hepatocellular carcinoma [35, 36]. Our data demonstrate that colon cancer cells rely on WDR5 for increased proliferation and cell survival as depletion of WDR5 reduced cell viability. Other groups have demonstrated similar findings and demonstrated that WDR5 is required for cell survival and proliferation in leukemia [37], prostate [15], bladder [38] breast [39], and pancreatic cancer [40].

In general, the mechanism by which WDR5 supports cancer cells has been shown to be through increased target gene expression. For example, WDR5 has been shown to promote EMT by promoting mesenchymal gene activation [41] and binding to ZNF407 to promote colon cancer metastasis [42]. Depletion of WDR5 reduced ErbB2 expression and cooperated with trastuzumab or chemotherapy to reduce ErbB2-positive breast cancer cell growth [39]. WDR5 has been shown to cooperate with HOTTIP to promote HOXA9 in prostate and pancreatic cancer [43, 44] and HOXA13 expression in esophageal and gastric cancer cells by increasing H3K4Me3 on their promoters [45, 46]. In bladder and gastric cancer, WDR5 increases the transcription of multiple cyclin proteins and stem cell-associated genes via increased H3K4Me3 [35, 38, 47–49]. Our data demonstrate that this is also likely the case in colon cancer cells, as WDR5 depletion caused global H3K4Me3 levels to decrease, which is believed to affect target gene transcription.

In conjunction with the findings that WDR5 is overexpressed and required in cancer, WDR5 has been shown to physically interact with Myc and promote target recognition contributing to tumorigenesis [50–55]. Interestingly, a study using patient-derived xenografts of pancreatic cancer demonstrated the WDR5:Myc interaction in vivo and showed this interaction prevented DNA damage accumulation [55]. Two other reports indicated that WDR5 regulated DNA replication and chromosomal polyploidy [56] as well as regulated abscission through localization to the midbody [57].

Our data demonstrated that, in colon cancer, WDR5 depletion induced a robust increase in γH2AX levels representative of an increase in DNA damage, which suggests WDR5 is contributing to DNA fidelity possibly through one of the previously described mechanisms. The contribution of WDR5 to DNA fidelity may or may not be independent of its role in the WRAD subcomplex as RBBP5 did not affect viability in a panel of colon cancer cells. However, there are multiple reports suggesting that depletion of MLL1 and MLL2 induce DNA damage as well as WDR5 suggesting a potential connection between the increased DNA damage following WDR5 depletion and its role in the COMPASS complex [5–10].

Resolution of γH2AX is thought to occur through exchange of γH2AX with dephosphorylated H2AX with subsequent dephosphorylation of the released γH2AX by phosphatases. One mechanism by which this occurs is following H3K4 and H3K36 methylation by metnase, a protein that contains a SET domain and is a potential binding partner of WDR5 [58]. Metnase also promoted non-homologous end-joining, restart of stalled replication forks, resolution of γH2AX, and knockdown increased sensitivity to ionizing radiation [58]. WDR5 itself has been shown to promote the incorporation of H2AZ to promote global transcription [59] suggesting a potential mechanism where WDR5 regulates cell cycle progression through increased transcription (H2AZ incorporation) and release of cell cycle checkpoints (removal and dephosphorylation of γH2AX).

Our data demonstrated increased sensitivity to radiation, particularly in SW620 and RKO colon cancer cells. While the HCT116 cells demonstrated increased γH2AX following WDR5 depletion, WDR5 depletion alone was sufficient to increase γH2AX to the same level seen with the addition of radiation. Relative to the other cell lines, HCT116 cells demonstrated the highest induction of γH2AX with WDR5 depletion alone. This could be a result of the high level of genomic instability in these cells. This, in combination with the additive effect of WDR5 depletion following radiation-induced DNA damage, suggests WDR5 is particularly required in cells following DNA damage.

Overall, WDR5 depletion demonstrated a more robust phenotype than OICR-9429 treatment. Several factors could contribute to this disparity, but two likely possibilities are that either WDR5 plays a role independent of the COMPASS complex that is not inhibited by OICR-9429 treatment or mutations in WDR5 or other COMPASS components limit the efficacy of OICR-9429. Consistent with the second possibility, cells with limited mutations in WDR5 and KMT2/MLL proteins had increased sensitivity to both WDR5 depletion and OICR-9429. This could be because that without mutations in KMT2/MLL components, the KMT2/MLL proteins require COMPASS complex formation in order to function to methylate H3K4 as they have very little enzymatic activity alone. Cells containing WDR5 mutations could also be less sensitive to OICR-9429 as mutations could reduce the affinity of the drug for WDR5. Additional studies on the effect of mutations in KMT2/MLL proteins and WDR5 will provide further understanding of the role of WDR5 and the COMPASS complex in cancer. Further studies are also needed to fully distinguish if the role of WDR5 is a result of its contribution to the COMPASS complex, is due to an alternative mechanism, or a combination of multiple mechanisms.

## Conclusions

This study demonstrated WDR5 is highly overexpressed in colon cancer cells and is essential for colon cancer cell viability. We further show that depletion of WDR5 sensitizes cells to irradiation. Together, these data demonstrate a clear role for this protein in colon cancer. Treatment with OICR-9429 was particularly efficacious in SW620 and T84 cells; however, in other colon cancer cell lines, OICR-9429 was less effective than direct RNAi-mediated depletion of WDR5. Interestingly, the cells that were the most sensitive to OICR-9429 treatment had the fewest mutations in components of the COMPASS complex, suggesting a potential role for WDR5 inhibition particularly in tumors without KMT2/MLL or WDR5 mutations.

## Additional file

Additional file 1: **Table S1.** Frequency of KMT2/MLL Mutations in Colon Cancer Cell Lines. **Table S2.** Sequences of individual siRNA and shRNA duplexes. **Table S3.** Sequences of qPCR primers. **Figure S1.** Validation of four oligos used for RNAi-mediated WDR5 depletion. **Figure S2.** Drug dose response curve for OICR-9429 in HCT116 cells. **Figure S3.** Representative propidium iodide cell cycle analysis following WDR5 depletion or OICR-9429 treatment in three colon cancer cell lines. **Figure S4.** Evaluation of apoptosis via Annexin V/PI staining following WDR5 depletion or OICR-9429 treatment in three colon cancer cell lines. **Figure S5.** RBBP5 depletion does not affect cell viability in a panel of colon cancer cell lines. (PDF 394 kb)

## Abbreviations

COAD: Colon Adenocarcinoma
COMPASS: SET/MLL COMplex of Proteins ASsociated with Set1
COSMIC: Catalogue of Somatic Mutations in Cancer
DDR: DNA-damage response
H3K4: Histone 3 Lysine 4
H3K4Me1–3: mono-, di-, or tri-methylation of H3K4
HCEC: Human Colon Epithelial Cells
PI: Propidium Iodide
TCGA: The Cancer Genome Atlas
WRAD: WDR5, RBBP5, ASH2L, and DPY30

## References

[1] Li BE, Ernst P (2014). Two decades of leukemia oncoprotein epistasis: the MLL1 paradigm for epigenetic deregulation in leukemia. Exp Hematol.

[2] Yang W, Ernst P (2017). Distinct functions of histone H3, lysine 4 methyltransferases in normal and malignant hematopoiesis. Curr Opin Hematol.

[3] Shilatifard A (2012). The COMPASS family of histone H3K4 methylases: mechanisms of regulation in development and disease pathogenesis. Annu Rev Biochem.

[4] Ford DJ, Dingwall AK (2015). The cancer COMPASS: navigating the functions of MLL complexes in cancer. Cancer genetics.

[5] Benayoun BA, Pollina EA, Ucar D, Mahmoudi S, Karra K, Wong ED, Devarajan K, Daugherty AC, Kundaje AB, Mancini E (2014). H3K4me3 breadth is linked to cell identity and transcriptional consistency. Cell.

[6] Chen K, Chen Z, Wu D, Zhang L, Lin X, Su J, Rodriguez B, Xi Y, Xia Z, Chen X (2015). Broad H3K4me3 is associated with increased transcription elongation and enhancer activity at tumor-suppressor genes. Nat Genet.

[7] Ali A, Veeranki SN, Chinchole A, Tyagi S (2017). MLL/WDR5 complex regulates Kif2A localization to ensure chromosome Congression and proper spindle assembly during mitosis. Dev Cell.

[8] Kantidakis T, Saponaro M, Mitter R, Horswell S, Kranz A, Boeing S, Aygun O, Kelly GP, Matthews N, Stewart A (2016). Mutation of cancer driver MLL2 results in transcription stress and genome instability. Genes Dev.

[9] Esposito MT, Zhao L, Fung TK, Rane JK, Wilson A, Martin N, Gil J, Leung AY, Ashworth A, So CW (2015). Synthetic lethal targeting of oncogenic transcription factors in acute leukemia by PARP inhibitors. Nat Med.

[10] Dawkins JB, Wang J, Maniati E, Heward JA, Koniali L, Kocher HM, Martin SA, Chelala C, Balkwill FR, Fitzgibbon J (2016). Reduced expression of histone Methyltransferases KMT2C and KMT2D correlates with improved outcome in pancreatic ductal adenocarcinoma. Cancer Res.

[11] Guo C, Chen LH, Huang Y, Chang CC, Wang P, Pirozzi CJ, Qin X, Bao X, Greer PK, McLendon RE (2013). KMT2D maintains neoplastic cell proliferation and global histone H3 lysine 4 monomethylation. Oncotarget.

[12] Zhang C, Song C, Liu T, Tang R, Chen M, Gao F, Xiao B, Qin G, Shi F, Li W (2017). KMT2A promotes melanoma cell growth by targeting hTERT signaling pathway. Cell Death Dis.

[13] Mo R, Rao SM, Zhu YJ (2006). Identification of the MLL2 complex as a coactivator for estrogen receptor alpha. J Biol Chem.

[14] Toska E, Osmanbeyoglu HU, Castel P, Chan C, Hendrickson RC, Elkabets M, Dickler MN, Scaltriti M, Leslie CS, Armstrong SA (2017). PI3K pathway regulates ER-dependent transcription in breast cancer through the epigenetic regulator KMT2D. Science.

[15] Kim JY, Banerjee T, Vinckevicius A, Luo Q, Parker JB, Baker MR, Radhakrishnan I, Wei JJ, Barish GD, Chakravarti D (2014). A role for WDR5 in integrating threonine 11 phosphorylation to lysine 4 methylation on histone H3 during androgen signaling and in prostate cancer. Mol Cell.

[16] Malik R, Khan AP, Asangani IA, Cieslik M, Prensner JR, Wang X, Iyer MK, Jiang X, Borkin D, Escara-Wilke J (2015). Targeting the MLL complex in castration-resistant prostate cancer. Nat Med.

[17] Mouradov D, Sloggett C, Jorissen RN, Love CG, Li S, Burgess AW, Arango D, Strausberg RL, Buchanan D, Wormald S (2014). Colorectal cancer cell lines are representative models of the main molecular subtypes of primary cancer. Cancer Res.

[18] Roig AI, Eskiocak U, Hight SK, Kim SB, Delgado O, Souza RF, Spechler SJ, Wright WE, Shay JW (2010). Immortalized epithelial cells derived from human colon biopsies express stem cell markers and differentiate in vitro. Gastroenterology.

[19] Grebien F, Vedadi M, Getlik M, Giambruno R, Grover A, Avellino R, Skucha A, Vittori S, Kuznetsova E, Smil D (2015). Pharmacological targeting of the Wdr5-MLL interaction in C/EBPalpha N-terminal leukemia. Nat Chem Biol.

[20] Jiang P, Du W, Mancuso A, Wellen KE, Yang X (2013). Reciprocal regulation of p53 and malic enzymes modulates metabolism and senescence. Nature.

[21] Getlik M, Smil D, Zepeda-Velazquez C, Bolshan Y, Poda G, Wu H, Dong A, Kuznetsova E, Marcellus R, Senisterra G (2016). Structure-based optimization of a small molecule antagonist of the interaction between WD repeat-containing protein 5 (WDR5) and mixed-lineage leukemia 1 (MLL1). J Med Chem.

[22] Dias J, Van Nguyen N, Georgiev P, Gaub A, Brettschneider J, Cusack S, Kadlec J, Akhtar A (2014). Structural analysis of the KANSL1/WDR5/KANSL2 complex reveals that WDR5 is required for efficient assembly and chromatin targeting of the NSL complex. Genes Dev.

[23] Ruthenburg AJ, Wang W, Graybosch DM, Li H, Allis CD, Patel DJ, Verdine GL (2006). Histone H3 recognition and presentation by the WDR5 module of the MLL1 complex. Nat Struct Mol Biol.

[24] Dou Y, Milne TA, Ruthenburg AJ, Lee S, Lee JW, Verdine GL, Allis CD, Roeder RG (2006). Regulation of MLL1 H3K4 methyltransferase activity by its core components. Nat Struct Mol Biol.

[25] Couture JF, Collazo E, Trievel RC (2006). Molecular recognition of histone H3 by the WD40 protein WDR5. Nat Struct Mol Biol.

[26] Han Z, Guo L, Wang H, Shen Y, Deng XW, Chai J (2006). Structural basis for the specific recognition of methylated histone H3 lysine 4 by the WD-40 protein WDR5. Mol Cell.

[27] Wysocka J, Swigut T, Xiao H, Milne TA, Kwon SY, Landry J, Kauer M, Tackett AJ, Chait BT, Badenhorst P (2006). A PHD finger of NURF couples histone H3 lysine 4 trimethylation with chromatin remodelling. Nature.

[28] Wysocka J, Swigut T, Milne TA, Dou Y, Zhang X, Burlingame AL, Roeder RG, Brivanlou AH, Allis CD (2005). WDR5 associates with histone H3 methylated at K4 and is essential for H3 K4 methylation and vertebrate development. Cell.

[29] Wang KC, Yang YW, Liu B, Sanyal A, Corces-Zimmerman R, Chen Y, Lajoie BR, Protacio A, Flynn RA, Gupta RA (2011). A long noncoding RNA maintains active chromatin to coordinate homeotic gene expression. Nature.

[30] Xu Z, Gao X, He Y, Ju J, Zhang M, Liu R, Wu Y, Ma C, Ma C, Lin Z (2012). Synergistic effect of SRY and its direct target, WDR5, on Sox9 expression. PLoS One.

[31] Ang YS, Tsai SY, Lee DF, Monk J, Su J, Ratnakumar K, Ding J, Ge Y, Darr H, Chang B (2011). Wdr5 mediates self-renewal and reprogramming via the embryonic stem cell core transcriptional network. Cell.

[32] Yang YW, Flynn RA, Chen Y, Qu K, Wan B, Wang KC, Lei M, Chang HY (2014). Essential role of lncRNA binding for WDR5 maintenance of active chromatin and embryonic stem cell pluripotency. eLife.

[33] Migliori V, Mapelli M, Guccione E (2012). On WD40 proteins: propelling our knowledge of transcriptional control?. Epigenetics.

[34] Migliori V, Muller J, Phalke S, Low D, Bezzi M, Mok WC, Sahu SK, Gunaratne J, Capasso P, Bassi C (2012). Symmetric dimethylation of H3R2 is a newly identified histone mark that supports euchromatin maintenance. Nat Struct Mol Biol.

[35] Dai X, Guo W, Zhan C, Liu X, Bai Z, Yang Y (2015). WDR5 expression is prognostic of breast Cancer outcome. PLoS One.

[36] Quagliata L, Matter MS, Piscuoglio S, Arabi L, Ruiz C, Procino A, Kovac M, Moretti F, Makowska Z, Boldanova T (2014). Long noncoding RNA HOTTIP/HOXA13 expression is associated with disease progression and predicts outcome in hepatocellular carcinoma patients. Hepatology.

[37] Ge Z, Song EJ, Kawasawa YI, Li J, Dovat S, Song C (2016). WDR5 high expression and its effect on tumorigenesis in leukemia. Oncotarget.

[38] Chen X, Xie W, Gu P, Cai Q, Wang B, Xie Y, Dong W, He W, Zhong G, Lin T (2015). Upregulated WDR5 promotes proliferation, self-renewal and chemoresistance in bladder cancer via mediating H3K4 trimethylation. Sci Rep.

[39] Mungamuri SK, Murk W, Grumolato L, Bernstein E, Aaronson SA (2013). Chromatin modifications sequentially enhance ErbB2 expression in ErbB2-positive breast cancers. Cell Rep.

[40] Cheng Y, Jutooru I, Chadalapaka G, Corton JC, Safe S (2015). The long non-coding RNA HOTTIP enhances pancreatic cancer cell proliferation, survival and migration. Oncotarget.

[41] Wu MZ, Tsai YP, Yang MH, Huang CH, Chang SY, Chang CC, Teng SC, Wu KJ (2011). Interplay between HDAC3 and WDR5 is essential for hypoxia-induced epithelial-mesenchymal transition. Mol Cell.

[42] Tan X, Chen S, Wu J, Lin J, Pan C, Ying X, Pan Z, Qiu L, Liu R, Geng R (2017). PI3K/AKT-mediated upregulation of WDR5 promotes colorectal cancer metastasis by directly targeting ZNF407. Cell Death Dis.

[43] Malek R, Gajula RP, Williams RD, Nghiem B, Simons BW, Nugent K, Wang H, Taparra K, Lemtiri-Chlieh G, Yoon AR (2017). TWIST1-WDR5-Hottip regulates Hoxa9 chromatin to facilitate prostate Cancer metastasis. Cancer Res.

[44] Fu Z, Chen C, Zhou Q, Wang Y, Zhao Y, Zhao X, Li W, Zheng S, Ye H, Wang L (2017). LncRNA HOTTIP modulates cancer stem cell properties in human pancreatic cancer by regulating HOXA9. Cancer Lett.

[45] Lin C, Wang Y, Wang Y, Zhang S, Yu L, Guo C, Xu H (2017). Transcriptional and posttranscriptional regulation of HOXA13 by lncRNA HOTTIP facilitates tumorigenesis and metastasis in esophageal squamous carcinoma cells. Oncogene.

[46] Chang S, Liu J, Guo S, He S, Qiu G, Lu J, Wang J, Fan L, Zhao W, Che X (2016). HOTTIP and HOXA13 are oncogenes associated with gastric cancer progression. Oncol Rep.

[47] Chen X, Gu P, Li K, Xie W, Chen C, Lin T, Huang J (2015). Gene expression profiling of WDR5 regulated genes in bladder cancer. Genomics data.

[48] Sun W, Guo F, Liu M. Up-regulated WDR5 promotes gastric cancer formation by induced cyclin D1 expression. J Cell Biochem. 2017;119(4)3304–3316. 10.1002/jcb.26491 29125890

[49] Sun TT, He J, Liang Q, Ren LL, Yan TT, Yu TC, Tang JY, Bao YJ, Hu Y, Lin Y (2016). LncRNA GClnc1 promotes gastric carcinogenesis and may act as a modular scaffold of WDR5 and KAT2A complexes to specify the histone modification pattern. Cancer discovery.

[50] Sun Y, Bell JL, Carter D, Gherardi S, Poulos RC, Milazzo G, Wong JW, Al-Awar R, Tee AE, Liu PY (2015). WDR5 supports an N-Myc transcriptional complex that drives a Protumorigenic gene expression signature in neuroblastoma. Cancer Res.

[51] Thomas LR, Wang Q, Grieb BC, Phan J, Foshage AM, Sun Q, Olejniczak ET, Clark T, Dey S, Lorey S (2015). Interaction with WDR5 promotes target gene recognition and tumorigenesis by MYC. Mol Cell.

[52] Thomas LR, Foshage AM, Weissmiller AM, Tansey WP (2015). The MYC-WDR5 Nexus and Cancer. Cancer Res.

[53] Ullius A, Luscher-Firzlaff J, Costa IG, Walsemann G, Forst AH, Gusmao EG, Kapelle K, Kleine H, Kremmer E, Vervoorts J (2014). The interaction of MYC with the trithorax protein ASH2L promotes gene transcription by regulating H3K27 modification. Nucleic Acids Res.

[54] Lorenzin F, Benary U, Baluapuri A, Walz S, Jung LA, von Eyss B, Kisker C, Wolf J, Eilers M, Wolf E. Different promoter affinities account for specificity in MYC-dependent gene regulation. eLife. 2016;5:e15161. 10.7554/eLife.15161 PMC4963202 27460974

[55] Carugo A, Genovese G, Seth S, Nezi L, Rose JL, Bossi D, Cicalese A, Shah PK, Viale A, Pettazzoni PF (2016). In Vivo Functional Platform Targeting Patient-Derived Xenografts Identifies WDR5-Myc Association as a Critical Determinant of Pancreatic Cancer. Cell Rep.

[56] Blumenthal DT, Dvir A, Lossos A, Tzuk-Shina T, Lior T, Limon D, Yust-Katz S, Lokiec A, Ram Z, Ross JS (2016). Clinical utility and treatment outcome of comprehensive genomic profiling in high grade glioma patients. J Neuro-Oncol.

[57] Bailey JK, Fields AT, Cheng K, Lee A, Wagenaar E, Lagrois R, Schmidt B, Xia B, Ma D (2015). WD repeat-containing protein 5 (WDR5) localizes to the midbody and regulates abscission. J Biol Chem.

[58] De Haro LP, Wray J, Williamson EA, Durant ST, Corwin L, Gentry AC, Osheroff N, Lee SH, Hromas R, Nickoloff JA (2010). Metnase promotes restart and repair of stalled and collapsed replication forks. Nucleic Acids Res.

[59] Kim K, Punj V, Choi J, Heo K, Kim JM, Laird PW, An W (2013). Gene dysregulation by histone variant H2A.Z in bladder cancer. Epigenetics Chromatin.

