# Supplementary material for: WDR5 supports colon cancer cells by promoting methylation of H3K4 and suppressing DNA damage
Source: BMC Cancer. 2018 Jun 20;18:673. doi: 10.1186/s12885-018-4580-6 (PMC6011590; doi:10.1186/s12885-018-4580-6)
Supplement: Supplementary file 1 — Table S1. Frequency of KMT2/MLL Mutations in Colon Cancer Cell Lines. Table S2. Sequences of individual siRNA and shRNA duplexes. Table S3. Sequences of qPCR primers. Figure S1. Validation of four oligos used for RNAi-mediated WDR5 depletion. Figure S2. Drug dose response curve for OICR-9429 in HCT116 cells. Figure S3. Representative propidium iodide cell cycle analysis following WDR5 depletion or OICR-9429 treatment in three colon cancer cell lines. Figure S4. Evaluation of apoptosis via Annexin V/PI staining following WDR5 depletion or OICR-9429 treatment in three colon cancer cell lines. Figure S5. RBBP5 depletion does not affect cell viability in a panel of colon cancer cell lines. (PDF 394 kb) [file 12885_2018_4580_MOESM1_ESM.pdf]

Additional File 1.pdf includes:

- Table S1: Frequency of KMT2/MLL Mutations in Colon Cancer Cell Lines
- Table S2: Sequences of individual siRNA and shRNA duplexes
- Table S3: Sequences of qPCR primers
- Figure S1: Validation of four oligos used for RNAi-mediated WDR5 depletion
- Figure S2: Drug dose response curve for OICR-9429 in HCT116 cells
- Figure S3: Representative propidium iodide cell cycle analysis following WDR5 depletion or OICR-9429 treatment in three colon cancer cell lines
- Figure S4: Evaluation of apoptosis via Annexin V/PI staining following WDR5 depletion or OICR-9429 treatment in three colon cancer cell lines
- Figure S5: RBBP5 depletion does not affect cell viability in a panel of colon cancer cell lines

Cell Line	KMT2A	KMT2B	KMT2C	KMT2D	KMT2F	KMT2G	WDR5
HCT116		D550fs[2]<br>G1881fs[1]<br>L2136fs[2]	Q419R[1]<br>I1344fs[1,2]	V160M[2]<br>R2173fs[1]<br>R2443fs[1,2]		G1578fs[2]	Y260H[1,2]
HCT15	T771fs[2]<br>S1062Y[1,2]<br>V2178A[1]	R226W[2]<br>R1125H[2]<br>A1989T[2]	G3438D[1,2]<br>S3543N[1,2]	P1152H[2]<br>E1244D[1]<br>E1517D[1,2]<br>P1931H[2]		D159N[2]<br>Q780H[2]	
SW480				D1633N[2]			
SW620		G273S[2]<br>G292V[1,2]					
RKO	T771fs[2]<br>S873fs[2]<br>D877fs[1]	A905T[2]<br>R1579H[2]<br>D1819fs[2]<br>P1823fs[1]	G2636C[1]<br>R3853W[2]	R916H[1]<br>R1189H[1,2]<br>L1327P[1]<br>L1600P[1,2]<br>G1960fs[2]<br>P2550fs[2]<br>G3465*[1,2]<br>L4516P[1]<br>R4964fs[2]<br>L5056P[1,2]	A1325T[2]	T1657A[2]	V217Ins[1]
LoVo		R525W[1,2]<br>P2255L[2]	C1013R[1]<br>E1313K[1]	H77fs[1,2]<br>P647fs[1,2]<br>P648fs[1,2]<br>T3548I[2]		V1404I[2]	G277D[1,2]
T84				None[1,2]			

Table S1: Frequency of KMT2/MLL Mutations in Colon Cancer Cell Lines

[1]=COSMIC
[2]=Mouradov *et al*

Note: SW480 were not evaluated in COSMIC

Target	Item #	siRNA	Target Sequence
Human WDR5	L-013383	5	GACGAAAGCGUGAGGAUAU
		6	GUGGAAGAGUGACUGCUAA
		7	GACGUGAGCUCGGGCAAGU
		8	GAUGGAUCCUUGAUAGUUU
Human RBBP5	LU-012008	5	UAACACGGCAGAUCGAAUA
		6	UAUAGAACUUCAAGGAGUA
		7	GCAAUACCACAGCCAUUAA
		8	GAUGGAACUUUGGAUUGUA
Human Non-Targeting	D-001810	01	UGGUUUACAUGUCGACUAA

Table S2: Sequences of individual siRNA and shRNA duplexes

Target	Accession	Amplicon Length	Exons	Tm	F Primers 5' -> 3'	R primer 5' -> 3'
Human WDR5	NM_052821	135 bp	8-10	58ºC	ATGCGACAGAGACCATCATAG	CGTGAGGATATGGGATGTGAA

Table S3: Sequences of qPCR primers

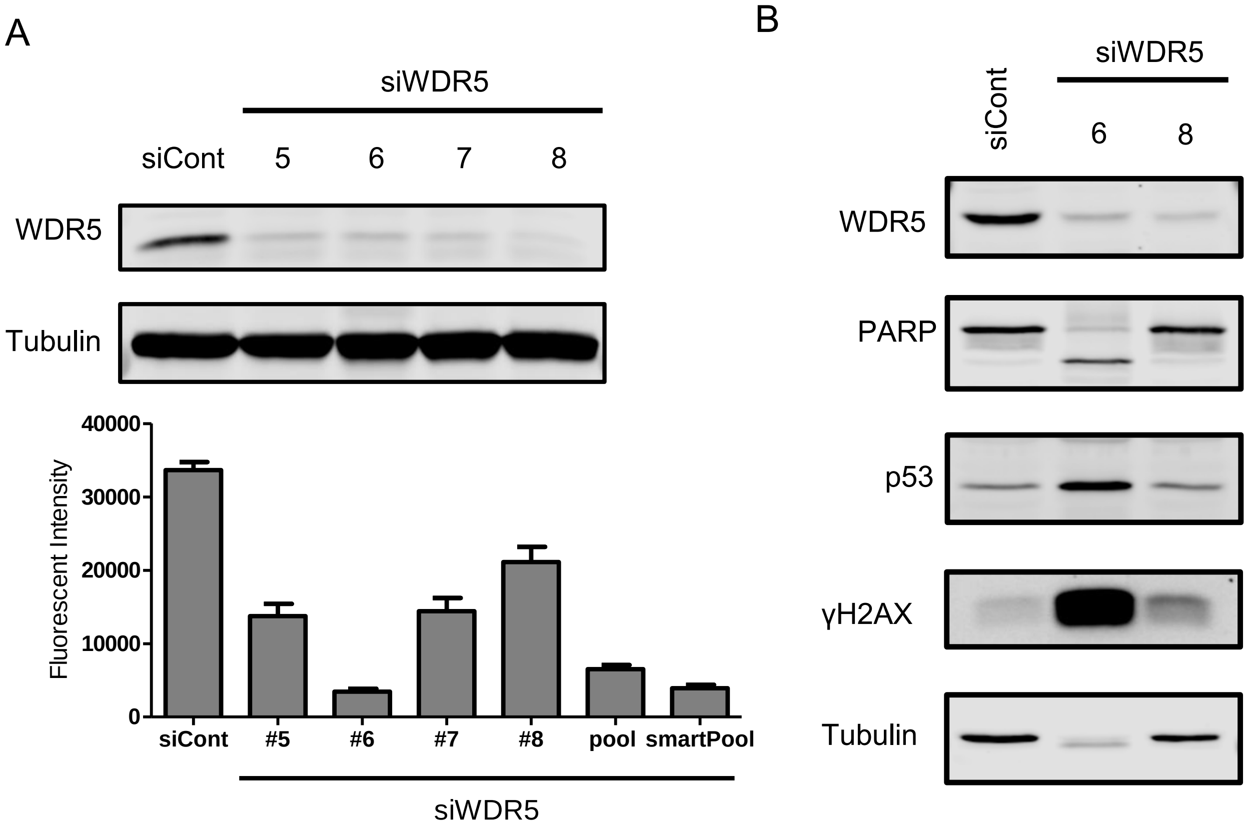

C

Oligo #6: GUGGAAGAGUGACUGCUAA

Figure S1: Evaluation of individual WDR5 siRNA. (A) Immunoblot of WDR5 and viability as measured by alamarBlue® following RNAi-mediated knockdown with individual siRNA oligos in HCT116 colon cancer cells. (B) Immunoblot of WDR5, PARP, p53, and γH2AX following RNAi-mediated WDR5 depletion using either oligo #6 or #8. (C) WDR5 #6 oligo sequence with nucleotides that match the ME1 sequence shown in red.

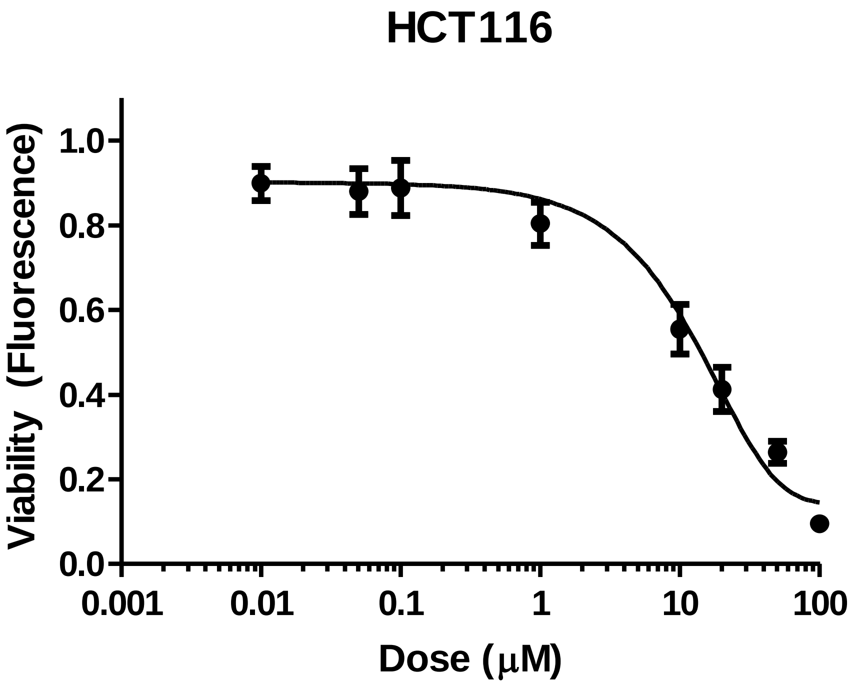

Figure S2: Drug dose response curve for OICR-9429 in HCT116 cells. Viability as measured by alamarBlue® following treatment with OICR-9429 at the specified doses for 48 hours in HCT116 colon cancer cells.

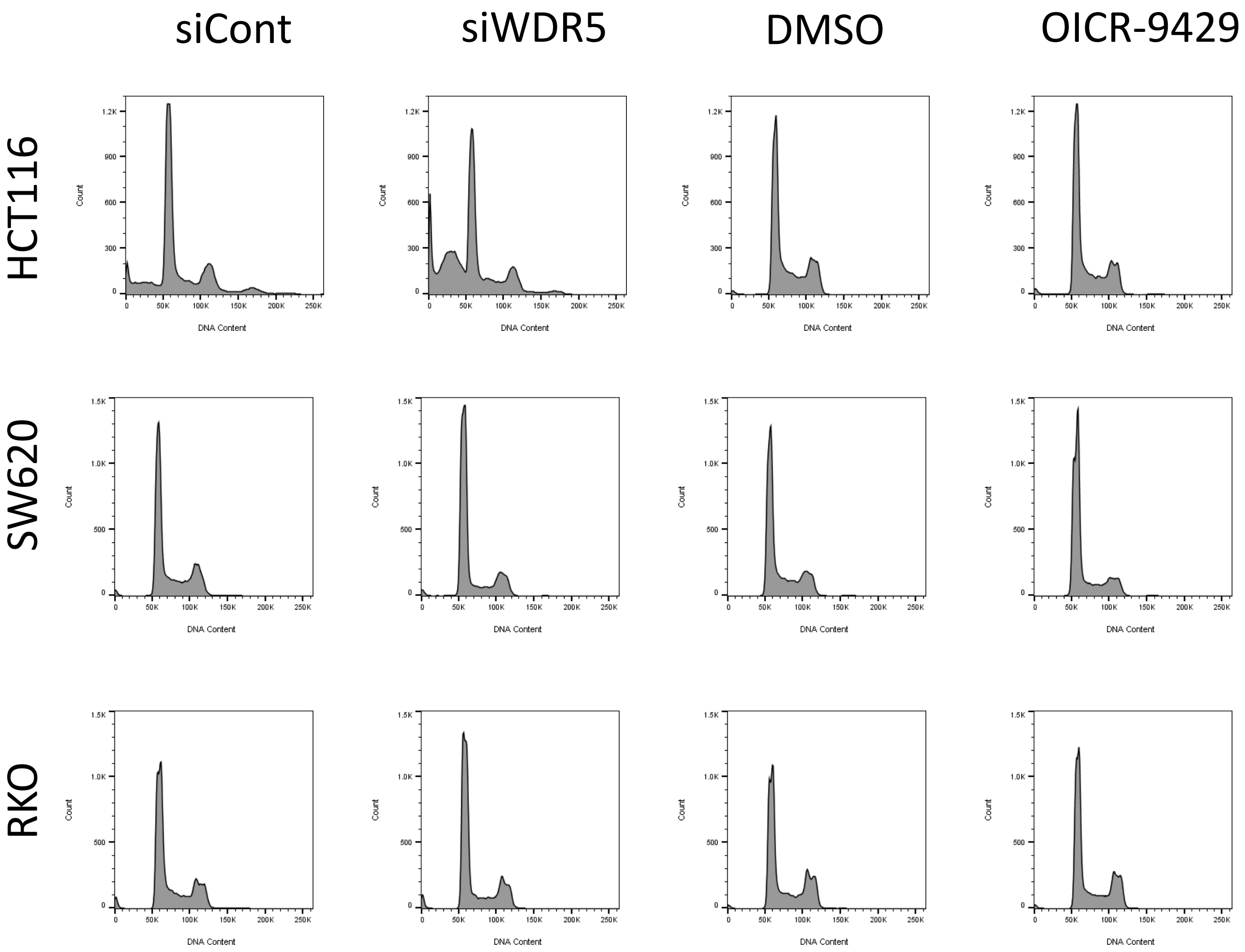

Figure S3: Representative flow cytometry results from propidium iodide cell cycle analysis in three colon cancer cell lines following RNAi-mediated WDR5 depletion for 72 hours (left) or 10 μM OICR-9429 treatment (right) for 48 hours.

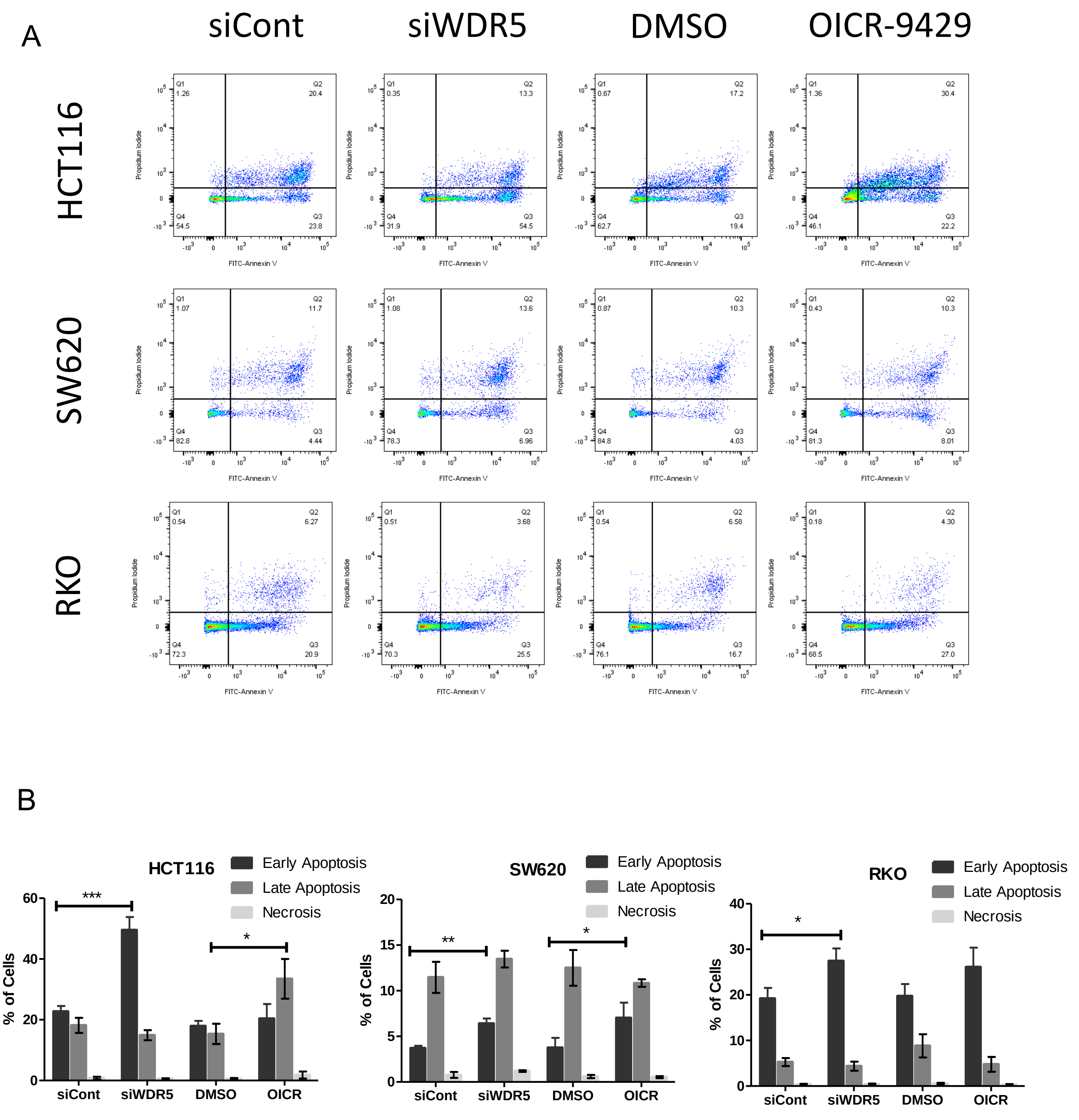

Figure S4: Evaluation of apoptosis following WDR5 depletion for 72 hours or 10 µM OICR-9429 treatment for 48 hours in three colon cancer cell lines. (A) Representative flow cytometry results with (B) quantification from three biological replicates in three colon cancer cell lines.

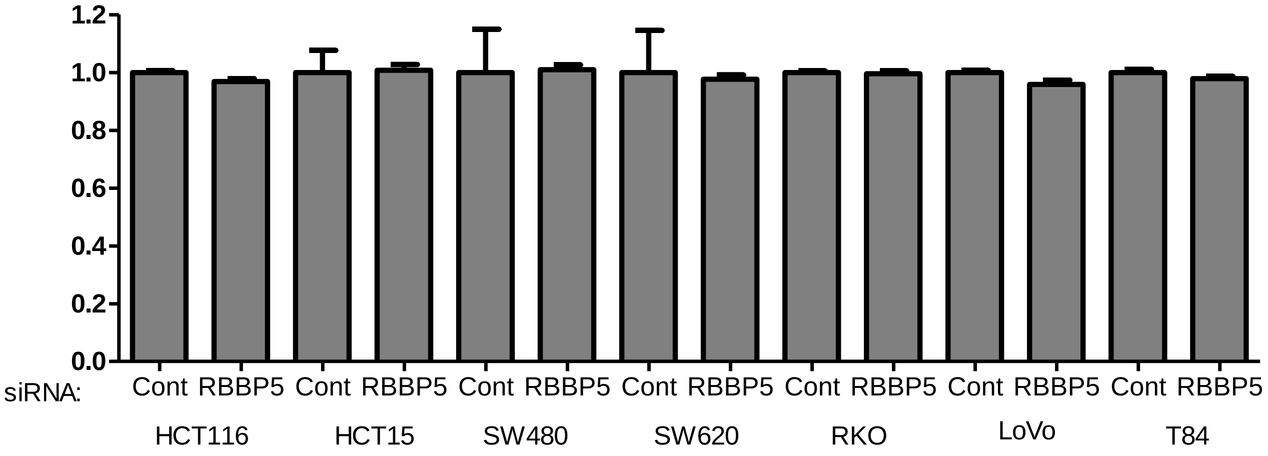

Figure S5: RBBP5 depletion does not affect cell viability in a panel of colon cancer cell lines. Cell viability in a panel of colon cancer cells as compared to HCECs following RNAi-mediated depletion of RBBP5. Viability was measured by alamarBlue® assays 72 hours after transfection.
